# Nurses’ expectations about the succession of leaders in the hospital context*

**DOI:** 10.1590/1518-8345.2833.3178

**Published:** 2019-10-07

**Authors:** Alyne Leite Gomes Nogueira, Denize Bouttelet Munari, Luana Cássia Miranda Ribeiro, Ana Lúcia Queiroz Bezerra, Lucieli Dias Pedreschi Chaves

**Affiliations:** 1 Universidade Federal de Goiás, Hospital das Clínicas, Goiânia, GO, Brazil.; 2 Universidade Federal de Goiás, Faculdade de Enfermagem, Goiânia, GO, Brazil.; 3 Universidade de São Paulo, Escola de Enfermagem de Ribeirão Preto, PAHO/WHO Collaborating Centre for Nursing Research Development, Ribeirão Preto, SP, Brazil.

**Keywords:** Leadership, Nurses, Nursing Staff, Professional Competence, Nursing Research, Nursing Supervisory, Liderança, Enfermeiras e Enfermeiros, Recursos Humanos de Enfermagem, Competência Profissional, Pesquisa em Enfermagem, Supervisão de Enfermagem, Liderazgo, Enfermeros, Competencia Profesional, Personal de Enfermería, Investigación en Enfermería, Supervisión de Enfermería

## Abstract

**Objective:**

to analyze the expectation of nurses about the succession of leaders in the hospital context.

**Method:**

a descriptive and exploratory study of a qualitative approach carried out with 36 nurses from a public university hospital selected using the snowball technique. The data collection was done through semi-structured interviews that were recorded and analyzed based on the assumptions of the Content Analysis technique, Thematic modality. Ethical criteria were adequately met.

**Results:**

the reports comprised two central categories, “Who Will Take My Place?” and “Potential and Openness to Plan the Succession of Leadership in Nursing,” the latter being divided into two subcategories: “Nursing leadership in the hospital five years from now” and “Strengths for the implementation of a leadership succession plan”.

**Conclusion:**

from the results, positive aspects of the diagnosis of the leadership profile in the institution that favor the development of succession planning were verified. In addition, it was possible to perceive the necessity of the development of this planning in the hospital and its importance for the succession of leadership in Nursing. In this context, this study presents itself with an innovative character for exposing a theme that aims to potentiate the future of Nursing.

## Abstract (pt)

**Objetivo:**

analisar a expectativa de enfermeiros acerca da sucessão de lideranças no contexto hospitalar.

**Método:**

estudo descritivo e exploratório, de abordagem qualitativa, realizado com 36 enfermeiros de um hospital público universitário selecionados a partir da técnica da bola de neve. A coleta de dados foi feita por meio de entrevistas semiestruturadas que foram gravadas e analisadas a partir dos pressupostos da técnica de Análise de Conteúdo, modalidade Temática. Os critérios éticos foram devidamente atendidos.

**Resultados:**

os relatos compuseram duas categorias centrais, “Quem vai ocupar o meu lugar?” e “Potencial e abertura para planejar a sucessão de liderança em Enfermagem”, essa última dividida em duas subcategorias: “A liderança de Enfermagem no hospital daqui a cinco anos” e “Fortalezas para a implementação de um plano de sucessão de lideranças”.

**Conclusão:**

a partir dos resultados, foram verificados aspectos positivos do diagnóstico do perfil da liderança existente na instituição que favorecem o desenvolvimento do planejamento de sucessão. Além disso, foi possível perceber a necessidade do desenvolvimento desse planejamento no hospital e sua importância para a sucessão de liderança em Enfermagem. Nesse contexto, este estudo apresenta-se com caráter inovador por expor uma temática que tem por objetivo potencializar o futuro da Enfermagem.

## Abstract (es)

**Objetivo:**

analizar la expectativa de enfermeros acerca de la sucesión de liderazgos en el contexto hospitalario.

**Método:**

estudio descriptivo exploratorio de abordaje cualitativo realizado con 36 enfermeros, en un hospital público universitario, seleccionados a partir de la técnica bola de nieve. La recogida de datos fue realizada por medio de entrevistas semiestructuradas que fueron grabadas y analizadas a partir de los presupuestos de la técnica de análisis de contenido, modalidad temática. Los criterios éticos fueron debidamente atendidos.

**Resultados:**

los relatos compusieron dos categorías centrales: “¿Quién va a ocupar mi lugar?” y “Potencial y abertura para planificar la sucesión de liderazgo en enfermería”, esa última dividida en dos subcategorías “El liderazgo de enfermería en el hospital dentro de cinco años” y “Fortalezas para la implementación de un plan de sucesión de liderazgos”.

**Conclusión:**

a partir de los resultados fueron verificados aspectos positivos del diagnóstico del perfil del liderazgo existente en las instituciones que favorecen el desarrollo de la planificación de la sucesión. Además de eso, fue posible percibir la necesidad del desarrollo de esa planificación en el hospital y su importancia para la sucesión de liderazgo en enfermería. En ese contexto, este estudio se presenta con carácter innovador por presentar una temática que tiene por objetivo potencializar el futuro de la enfermería.

## Introduction

The succession of leaderships is now a challenge in the management of human capital due to the shortage of professionals with profiles required to manage people, considering the diversity of skills required in the context of the world of work.

The absence of people capable of succeeding the leaders limits the growth and development of an organization. For the achievement of the leadership succession process, it is necessary to integrate, in strategic planning, the definition of skills for the professionalization of these future leaders^(^ 1 ^)^.

In this study, leadership is understood as the ability to influence people to their commitment to the purposes of an organization, identifying and valuing their talents ^(^ 2 ^)^. In this perspective, leadership does not concern positions, status or privileges, but rather a model to be followed by its moral and ethical examples. Thus, the leader has the mission of forming followers, seeking the best collective performance in producing results^(^ 2 ^)^. Leadership succession studies are modeled on leadership models that value the best performance of followers for organizational success and job satisfaction^(^ 3 ^)^.

The labor market has required that Nursing leaders be willing to update their knowledge and skills about leadership^(^ 4 ^-^ 5 ^)^, above all, seeking the development of styles that contemplate the urgent need to adapt to the current changes and needs of society^(^ 3 ^,^ 5 ^-^ 9 ^)^.

Nursing leaders are vital to health organizations^(^ 5 ^,^ 10 ^)^. Therefore, the preparation of nurses to assume a leadership position should be a concern of the training institutions and those who employ them ^(^ 4 ^,^ 8 ^-^ 11 ^)^. The development of nursing leadership requires specific strategies and programs, as well as evaluation processes and monitoring of learning^(^ 6 ^,^ 10 ^-^ 12 ^)^.

Studies show that organizations have devoted little attention to the development of leaders in Nursing^(^ 4 ^-^ 6 ^,^ 10 ^-^ 15 ^)^. The lack of preparation of the nurses for the succession of leaderships is pointed out as a problem to be faced by Nursing^(^ 8 ^,^ 10 ^-^ 18 ^)^. In turn, health organizations also have responsibilities to include, in their strategic planning, leadership-focused training programs. The preparation of new nurses is essential for filling vacancies for succession^(^ 16 ^,^ 18 ^)^.

This process can be organized through Succession Planning (SP), defined as a “strategic process that involves the identification, development and evaluation of individuals for future leadership positions, ensuring continuity of leadership within an organization”^(^ 14 ^)^. The SP allows key positions in organizations to be filled internally when they are vacant because of retirement, internal conflict or health leave, for example^(^ 19 ^)^. Using this strategy prevents key positions from becoming vulnerable^(^ 19 ^)^ and ensures the continuity of projects, culture and institutional achievements^(^ 17 ^)^.

Evidence of the importance of this issue stems from the aging of Nursing leaders noted by the imminent retirement of experienced nurses projected in the short and medium term^(^ 8 ^,^ 10 ^-^ 11 ^,^ 14 ^-^ 15 ^,^ 18 ^,^ 20 ^-^ 21 ^)^. In addition, the difficulty of recruiting new leaders has been evidenced in studies that point out little willingness of young people to take leadership positions^(^ 13 ^-^ 14 ^,^ 18 ^,^ 20 ^-^ 21 ^)^.

The characterization of the workforce in Nursing is composed of individuals of up to four generations still working together. These are represented by the Veterans / Traditionalists (1925 to 1945), who, though mostly retired, still act at some level in organizations; the Baby Boomers, who were born between 1946 and 1964, who are already able to retire, but some of them continue to act in leadership positions in organizations; the generation X (1965 to 1980) forms the strategic nucleus in the organizations and the generation Y / Millenium (1981 to 2000) is of young people recently admitted in the organizations^(^ 22 ^)^. This scenario indicates that the implementation of SP in organizations can prepare in advance new generations of leaders in Nursing^(^ 23 ^)^. It is also worth considering that the composition and coexistence between these generations can be limiting or potent for the succession process, depending on their conduct.

The justification for the accomplishment of this study is based on the literature review in which it was verified that the succession of leaderships is an innovative and incipient theme at the national level. Although Brazilian nurses have advanced in leadership studies, there are no researches with this focus, although some mention the importance of the nurse training process as a leader both in academic and practical contexts as part of their professional development^(^ 4 ^,^ 9 ^,^ 24 ^-^ 27 ^)^.

This research brings an innovative approach to Nursing leadership by awakening the need to develop, in a dynamic and systematized way, the formation of new leaderships in Nursing. This process can favor the future and the preservation of the legacy of generations of more experienced nurses, as well as the advancement of the profession and the accomplishment of research with another perspective on leadership and management. It is also hoped that the study will inspire other researchers in the search for strategies that favor the good coexistence between the generations of nurses in the different spaces where they work.

Thus, the objective of the study was to analyze the nurses’ expectations about the succession of leaders in the hospital context.

## Method

A descriptive and exploratory study with a qualitative approach, whose purpose is “to use non-quantitative methods to contribute with new knowledge and provide other perspectives in the health area”^(^ 28 ^)^. The qualitative research guide (*Consolidated criteria for reporting qualitative research (COREQ): a 32-item checklist for interviews and focus groups*)^(^ 28 ^)^ was used as reference in the planning and execution of the research. The research was carried out in a public university hospital of the Central-West region of Brazil, in the year 2016.

Participants were nurses in the exercise of management positions with at least one year of work in the institution. For the selection of participants, the snowball technique was used^(^ 29 ^)^ in which, at the end of the interview, each nurse indicated two others to participate in the study, and so on. The use of this criterion allowed that they had leaders representing all levels of performance. Those nurses who were indicated and, at the time of the interview, were separated from the activities for any reason were excluded.

The study sample was intentional, composed of 36 nurses, and, in order to delimit the number of participants, the data saturation criterion was used. This process was defined when we observed the repetition of perception about the same data^(^ 30 ^)^. The continuous process of analyzing the data of each interview ensured the consistency and robustness of the information.

To obtain the data, we used in-depth and semi-structured interviews that explored participants’ experiences and the meanings they attribute to the studied subject. The meetings for the collection were previously scheduled according to the availability of the participants. All the interviews were carried out in the hospital’s premises, in a place that allowed privacy, during working hours, with the institutional consent.

The questions that made up the interview script focused on the institution’s concern regarding the replacement of nursing leaderships; how nurses saw this leadership in the institution in the coming years and what their expectations and willingness to participate in a proposal for a leadership succession plan. In addition, questions related to the professional profile related to leadership.

The interviews were conducted by one of the authors and accompanied by the other, both with training in human relations dynamics and with expertise in qualitative research, in order to guarantee the fidelity of the information, the adequacy of the data collection route and the adequacy of the approach of participants.

In the first contact with the participants, the objective of the study was presented and the signing of the Free and Informed Consent Term (FICT) was requested. The interviews had an average duration of 40 minutes and were recorded in digital media with the acquiescence of the interviewees.

As the interviews were carried out, the information was transcribed for a preliminary analysis and identification of its saturation^(^ 30 ^)^. The data was submitted to Content Analysis, Thematic modality, by track, which is the process done from the clues given by the findings, leading to the construction of categories. The opposite of this process is called box analysis where the data is distributed in pre-determined analytical focus. In this process, the stages of pre-analysis, material exploration and treatment of results, inference and interpretation^(^ 31 ^)^. To assist in this phase, WebQDA 2016 software was used for data storage and coding. WebQDA was a tool that optimized data coding by allowing the system to feed the interview transcripts. These were arranged in the “internal sources” of the software, where each source represented the primary document of each interview transcribed. The resources of this tool enabled the creation of codes that indicated the units of analysis. After the sources were defined, the “tree codes” were created, so called because they allow branching. Each tree code was used to represent a category and its branches represented the subcategories, identifying the fragments of phrases, words or phrases that illustrated the codes created and represented the quotes of the participants’ speeches. In this way, analytical development generated two central categories, one of them being divided into two subcategories.

For the presentation of the data, codes have been created for each participant. In the elaboration of these codes, the first letter of the position that the nurse performs in the institution was assigned, numbering it according to the order of transcription. Thus, the nurses linked to the Nursing Board with the code “D” (D1 to D5) were grouped; to the heads of units, the code “C” (C1 to C8) was assigned; the code “L” (L1 to L7) and, to the supervisors, the “S” code (S1 to S16).

The research was developed respecting the Brazilian legislation for research with human beings and obtained a favorable opinion by ethics committee in research of the institution with protocol CAAE n. 54854716.9.0000.5078

## Results

Thirty-six nurses were enrolled in the study, 33 of whom were women and three were men. Table 1 illustrates data to understand a more detailed profile of these people.

**Table 1:** Characterization of the nurses participating in the study regarding the generation they represent, occupation hierarchy and leadership experience. Central-West Region, Brazil, 2016 (n = 36)

Characteristics	N Directors (05)	N Bosses (08)	N Leaders (07)	N Supervisors (16)	N Total (36)	%
**Generation**						
Y*			01	10	11	30.5
X^†^	02	05	05	05	17	47.2
Baby Boomers^‡^	03	03	01	01	08	22.2
**Position**						
Board of Directors	05				05	2.8%
Head of Unit		08			08	22.2%
Clinical Leader			07		07	19.4%
Clinical Supervisor				16	16	44.4%
**Did a specific course on leadership**						
Yes	02	04	03	02	11	30.6%
No	03	04	04	14	25	69.4%
**Had any leadership guidance to perform the role**						
Yes	03		03	09	15	41.7%
No	02	08	04	07	21	58.3%

* Y = Generation Y / Millennium (1981 to 2000);

† X = Generation X (1965 to 1980);

‡ Baby Boomers = Baby Boomers (1946 to 1964)

As regards the level of training of the 36 nurses, one (2.8%) graduated, 19 (52.8%) are specialists, 13 (36.1%) are masters, two (5.5%) are doctors and one (2.8%) are postdoctors. Among the specialists, four masters and, among the masters, five do doctorate.

**Figure 1:**
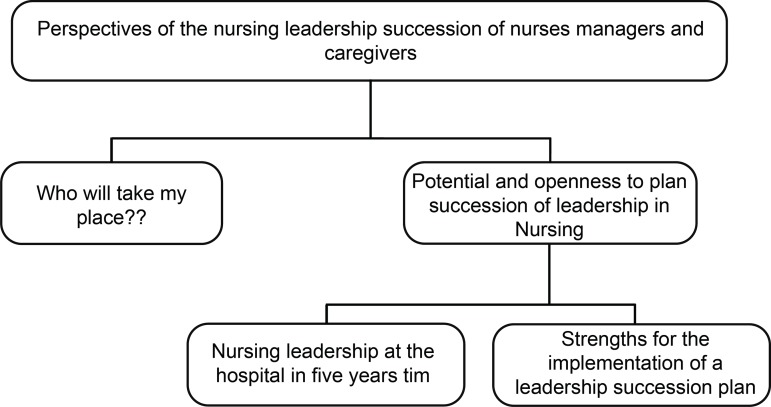
Categories and subcategories on the perspectives of the Nursing leadership succession of nurse managers and caregivers. Central-West Region, Brazil, 2016

The data from the individual interviews after being analyzed generated two thematic categories that will be presented in Figure 1.

Category 1, “Who will take my place?”, Expresses the awareness of the group of nurses regarding the issues related to the succession of nursing leaders in the hospital. This is a concern of the majority, regardless of the position. In this stratum, we still explore the reflections regarding the characteristics of the institution regarding the approximation of the outgoing nurses that occupy managerial positions and need to be replaced, as well as points out the need to prepare people to continue the service and routine of the hospital, as shown in Figure 2.

**Figure 2:** Narratives of nurses related to category 1 on the occupation of the leadership role in the hospital. Central-West Region, Brazil, 2016

Who will take my place?
*Both I and other Nursing leaders are close to retiring, it's only another five years, we sit down and talk about it, how it's going to be, who will replace us .... who will you lead?* (D1) *From the beginning, when I was a team, I wanted to work with new people, I already knew some people, and I already thought that my days inside the institution were counted, so I thought I needed people to continue the service, I just planted the seed, it needs to bear fruit and bear good fruit, so I had this concern of having a new team.* (D4) *Undoubtedly, it is a concern of all of us. It's very casual this conversation, a common conversation, when you need to change someone's place, when you need to go some to key places, you know, there is no formality in this sense, we talk about how it will be, but it's a question in our head .* (C1) *In several moments, my immediate boss arrives and makes direct notes, you will replace* *me.* (C4) *I realize that yes, we have our Head of Nursing, who is a born leader, when someone new arrives, she says thus: "you will be our successor," she has a lot of that concern.* (C8) *I talk a lot to the boys, because I want them to follow up, I'm jealous of the service. For him to be in good hands, then, all who have to want this.* (D4) *The built cannot be discarded, so when someone is going to retire, there is a concern with who will replace that person's place, now, prepare a person to be assuming, we do not have that hierarchy to do it here, I've never seen, until because of the same sizing.* (L7)

Category 2 refers to “Potential and openness to plan nursing succession leadership” and reflects the perspective of nurses to engage in leadership succession planning.

Of this second category, two subcategories emerged.

Subcategory 2.1, “Nursing leadership in the hospital five years from now,” shows how nurses have projected their perspectives for the coming years, indicating a certain hope in the potential successors, given the preparation of professionals and their potential for leadership.

The second subcategory 2.2, “Strengths for the implementation of a leadership succession plan,” demonstrates the availability of nurses to develop such a plan for the institution. The data is shown in Figure 3.

**Figure 3:** Narratives of nurses related to category 2 and their subcategories regarding the succession planning of leaders in the hospital. Central-West Region, Brazil, 2016

Potential and openness to plan succession of leadership in Nursing	
**Nursing leadership at the hospital in five years time**	*I think there will be a great demand for retirement, we had many at the beginning of this year and, five years from now, it must be bigger, I'll be in this group! This group of my group, we entered the public service in 90, we will be retired in five years or even less and you will assume and the prospect is good, as long as you are committed to the institution, at least knowledge, you are having, allied knowledge with commitment would be idea.* (D5) *I see it as a very positive point, some people will have to retire compulsorily and this succession will have to happen whether they want it or not. (C4)* *I think there has to be a total renewal in the nursing team, with training and courses, you have to have a lot of training to carry out a big hospital.* (S13) *I'm sure we'll have great nurses, very skilled in leadership, I think there are a lot of people with potential. (D2)* *I see with good perspective, are entering many new people with a very good profile. (D1)* *Today is beautiful to think, we have a group of doctorates in Nursing with a greater and better qualification, with a more effective professionalization, with this ability to convey, to plan, to lead. I very much believe that this will bear fruit, yes, I want to see this Nursing from five years to now totally different here, by the commitment of these doctors who are forming here and are the professionals that will replace us.* (C5) *I think it has to change, I think a lot of things get better when you see a new person doing it, I think it has to change, not that you have to change everybody, but there are some people who have been in the lead for a long time.* (S1)
**Strengths for the implementation of a leadership succession plan**	*If we think in a systematic way, we can have better educated leaders. (S14)* *I think it's necessary to think about it because otherwise we will face very serious problems in a few years.* (S6) *I think so, I think you should do it because being a leader today has changed your vision, in the old days, that was it, you said it and that's it, but today, no, to be a leader, you have to be able to lead, have an overview of the world, has changed a lot. If you develop this project here, the hospital has a lot to gain. (L2)* *We have several tools for this, we have a cadre of masters, doctors, post-doctors, we have an approach to the university, we find availability of university professors, we find the doors open. You have to have good will and I'm noticing that goodwill exists.* (L3) *I see that everyone is open to change too, and everyone wants the best for the future of the hospital, even those in management, they want the best too. (S11)* *For Nursing, it is essential to think about it, it is as if you were lighting a little light there and saying that we can leave, that the ground is prepared and this is very important. We cannot lose what we have achieved, from the moment I see that it will continue and that this planning will appear and that I will not have this fear that tomorrow, when I leave, that it is ready, is ready.* (D1) *This work you are doing is already opening up our awareness and concern. If you help make a model available, then there's going to be a building there, because this is happening to us now. If we can leave this product for everyone, as guidance, it will be too good because I believe in our profession, I believe in Nursing, I believe in the leadership power of those who are in here.* (C5)

## Discussion

The profile of the participants reflects the coexistence of three generations of nurses working together in the hospital, pointing to a similar picture to that described in the literature^(^ 22 ^-^ 23 ^)^. Representatives of the Baby Boomers and Generation X generation account for 69.4% of all study participants. Most of this group (52.7%) occupy the highest management positions, indicating possibilities of retirement in the short and medium term. This scenario alerts to the need to prepare new leaderships for the adequate supply of qualified leaders and future Nursing managers^(^ 16 ^-^ 17 ^,^ 19 ^)^.

Most of the representatives of generation X (12), because they already have a management function, can favor the development of leadership in the hospital. These professionals can both commit to their improvement in leadership as they may be possible mentors of younger professionals (generation Y).

The qualification of the participants shows a trend towards professional improvement since, of the nine professionals who are master’s or doctoral students, the majority is of the generation Y. The fact that the university hospital facilitates the proximity with the *stricto sensu* graduate courses in the university seems to stimulate the search for better qualification, even though most are still specialists (52.8%). This group of professionals adds to the institution a powerful intellectual capital and impresses on the organizational culture values for the permanent formation that should potentiate the institutional growth^(^ 23 ^)^. For this reason, the coexistence of this generation with the others requires institutional and relational strategies to be harnessed in its power, adding and not highlighting the differences.

The specific training for the exercise of leadership is not directly related to assumed management positions. Nurses in the top management positions did not take any specific course on leadership. This is not a reality of Brazil alone. Research conducted in the United States^(^ 10 ^)^ highlighted that Nursing leaders are generally promoted based on their clinical knowledge and effectiveness of their actions and not by having formal leadership education.

Likewise, the fact that most of the participants (58.3%) did not receive leadership guidance when they took office was highlighted. This data reveals that collaboration between the positions does not seem to be a formal concern in the institution. Despite the advances and evidences of leadership studies, this is still not seen as a competence to be developed^(^ 11 ^-^ 12 ^,^ 20 ^)^. In practice, it is considered as a “cream” characteristic and, for this reason, it is little valued as something to be learned. In organizations that have the SP implemented, collaboration is a key point and part of the organizational culture^(^ 20 ^)^. The awareness of nurse assistants and managers on the importance of effective communication and true spirit of collaboration is a key aspect in organizations that value the intergenerational work of nurses^(^ 3 ^,^ 11 ^,^ 16 ^)^.

The data of the professional profile and the leadership experience of the nurses point to an institutional scenario in the situation of imminent changes in the management positions occupied by these professionals. This signals an alert that attention is needed to overcome the difficulties of the loss of those with much experience, while at the same time indicating the need to form new leaderships. The speeches also indicate that it is possible to take advantage of this transition phase to prepare a new generation of leaders for the hospital, as will be seen below.

The data that illustrates the first category, “Who will take my place?”, Portray the feeling of concern for the future, not to lose what was built, above all, to preserve what has been won by each more experienced nurse, as if he could keep alive his efforts and personal dedication at work when he retired.

Although the hospital does not have a succession plan, it was verified in the nurses ‘statements explicit actions to map the nurses’ potential for the possible replacement of their positions, including direct indications. These indications, in some way, transfer to the younger ones a responsibility to maintain the achievements that the more experienced have achieved over the years. They also bring the burden of trying to preserve their working life histories in the hospital, leaving their personal marks.

In this sense, there are statements that express the concern to protect the patrimony that the Nursing built in the institutional scope: “I want them to follow up, I’m jealous of the service. For him to be in good hands, then, all who have to want this ...... I just planted the seed, it needs to bear fruit and bear good fruit, so I had this concern to have a new team “ (D4). When they refer that “The constructed cannot be discarded” (L7), an affective charge is brought about by the time spent in front of the post, showing concern for the continuity and preservation of what was constructed throughout their professional lives, as if the younger ones could keep their deeds alive and their effort.

The perspective of these professionals is that of who is in the management of the Nursing and carries the weight of this function, although it does not take into account what they think those who could be its successors. A study^(^ 3 ^)^ that discussed the perceptions of direct care nurses about the competencies needed for leadership showed how important the mutual listening process is between those in leadership positions and those on the front line of care. In its conclusion, the study makes clear the importance of strengthening communication among these professionals to better understand the potential of collaboration and recognition of the needs of professionals for better performance.

Experience in practice in the university context has shown that the preparation of new leaders is a process fraught with personal feelings and values. These, often, positively or negatively interfere in the transition of roles, particularly, for what each generation carries in its characteristics, values, beliefs and attitudes^(^ 22 ^)^. For this reason, understanding in depth how these processes happen can help in the preservation of an invaluable intellectual heritage.

The essence of this first category goes back to the idea that more experienced nurses care about who will take their places and whether this person will be able to maintain what was earned during a lifetime of work. Nurses representing the Baby Boomers generation, in particular, are professionals with a sense of belonging to their workplace, defend the institution, are proud of their achievements and their own trajectory^(^ 22 ^)^.

In this sense, the participants of the study, faced with questions about the succession of leaders, see that it is necessary to prepare people to give continuity to what was built. Thus, the contingent of professionals of generation Y could count on the experience of those of the generation Baby Boomers and the generation X for its formation in the next years. Studies of the effects of leadership development programs point to advantages of this process and also reinforce the importance of younger people knowing how the most experienced work and solve problems, although these moments are still limited by the lack of opportunities^(^ 6 ^,^ 8 ^,^ 10 ^-^ 11 ^)^.

The data for Table 2, which illustrates the second category, “Potential and openness to plan the succession of leadership in Nursing”, were organized into two subcategories.

The first one, “Nursing leadership in the hospital five years from now,” shows the perspective of nurses related to the succession of leadership in the medium term. These are directly linked to the possibility of renewal of the current leaders of the institution, due to the proximity of retirees of those who are more experienced.

Research highlights the importance of conducting an annual analysis of the leadership gaps in Nursing in the institutions, which allows the identification of predictable vacancies based on projected promotions in pensions and possible successors to take up these vacancies^(^ 32 ^)^.

The statements that illustrate the category, in general, bring a positive expectation to the hospital’s nursing leadership over the next five years. Among the aspects that seem to favor future and promising changes is the identification of the potential of the younger group and its training, given that many nurses of generation Y are masters or doctors or are still qualifying.

In this sense, this concern expressed in the nurses’ speech was also glimpsed that the replacement of the leaders is done considering the possibilities of training new nurses for the management and leadership. Studies^(^ 15 ^-^ 17 ^)^ point out that maintaining the internal intellectual capital of leaders enables the identification of high-potential nurses at all organizational levels to provide formal leadership development. Identifying high potential leaders and their strategic development can help ensure a suitable set of successors^(^ 32 ^)^.

The data also point to a possible renewal of people in positions that have long been occupied by the same professionals. This idea is accompanied by the prospect of improvement for the leadership in the institution due to the expectation placed on the nurses of generation X who already work in the hospital and the new generation of nurses of generation Y. However, it is important to consider that, rather than renewing the group of professionals, it is desirable to prepare them, as some statements.

In this sense, it is emphasized that international studies^(^ 6 ^,^ 11 ^,^ 20 ^,^ 23 ^,^ 32 ^-^ 34 ^)^ point out the need to develop a new generation of leading nurses based on more robust and innovative frameworks and methodologies. These references and methodologies, in general, have a strong influence on the principles of transformational leadership. These mobilize in leaders, among other aspects, better communication skills for the production of more egalitarian relations in the organizational context, facilitating the processing of changes and the achievement of results more satisfactory for all ^(^ 2 ^-^ 3 ^)^. Examples of successful nurse training initiatives in this sense can be found in the United States of America, for example, in several master’s degrees in leadership and management.

As the data from this study indicate, a survey indicates that generation Y professionals will assume leadership positions in the coming decades ^(^ 32 ^)^. For this reason, identifying high-potential internal intellectual capital is critical to the success of Succession Planning in Nursing^(^ 8 ^,^ 10 ^,^ 18 ^,^ 33 ^-^ 36 ^)^. This step is one of the elements to think of a succession planning that, among other aspects, also includes strategies of retention of the professionals in the services and development of orientation programs for the leadership. In addition, to be effective, it is the implanted system itself that reveals, shapes and sustains the succession planning of leaders, making it part of their organizational culture^(^ 37 ^)^.

In the second subcategory, “Strengths for the Implementation of a Leadership Succession Plan,” data were compiled that trace back to the idea that it is possible and necessary to begin organizing such planning at the institutional level.

A succession plan should be able to develop a leadership system with values based on principles to be achieved by professionals with openness to change and vision, courage to innovate and humility to learn continuously, since this is the challenge of the leader of the future^(^ 2 ^)^.

The data indicate a positive perspective of the nurses regarding the possible implantation of a SP in the hospital, based on the perception that there is openness of the institution and the nurses managers and assistants for this change. The support of the Nursing managers and the integration of the SP to the strategic planning of the institution is necessary for it to be successful^(^ 34 ^)^. An organizational culture of encouragement, leadership development and succession is essential for modeling new roles^(^ 38 ^)^.

The nurses emphasized the SP as a necessary conduct to systematize isolated actions made by one or another person. In addition, they consider that it can contribute to a better formation and qualification of leaders, to promote positive changes and the continuity of leadership in the institution.

Succession planning and leadership development enable the formation of a cadre of trained nurses to be potential successors, thus enabling continuity of leadership^(^ 13 ^,^ 21 ^,^ 32 ^-^ 33 ^)^. It also improves the work environment, patient care and nurses’ satisfaction^(^ 3 ^,^ 17 ^,^ 32 ^,^ 37 ^)^, being crucial for the future of the Nursing profession^(^ 34 ^)^. In the conclusion of an investigation conducted in the United States, the authors point out that “the way is clear: the nurse’s leadership competence influences job satisfaction and makes working environments healthy, which in turn influences the best way to get positive results from customers”^(^ 3 ^)^.

One of the lines highlights a nurse’s belief in the future and the need to systematize a hospital succession plan. This narrative reported to a recent study whose reflexive conclusion was: “The most significant contribution that leaders can make to the future of nursing is to develop their successors”^(^ 10 ^)^.

Thus, the SP should contribute to the training and development of skills and abilities necessary for the role of Nursing leaders able to act in a diversified, complex and highly competitive professional and organizational context.

The research had as a limitation the fact of focusing reality in a single scenario. In this sense, it is recommended that future studies can explore other contexts of public and private services to better understand the institutional needs, limitations and peculiarities of this process in the preparation of new leaderships.

The aspects of this research that advance in the knowledge of the subject and of Nursing are related to the fact of describing a proposal of vanguard on the leadership in Nursing and of awakening the need to develop systematized processes of formation of new leaderships in Nursing, that can enhance the future of Nursing, preserving the legacy of generations of more experienced nurses. Likewise, it highlights the potential and willingness of young nurses to implement SP.

## Conclusion

Based on the objective of analyzing nurses’ expectations about the succession of leaders in the hospital context, the findings of the study indicate a favorable scenario to consider the implementation of this process in the institution. This result is derived from a positive diagnosis of the existing leadership profile and the openness of professionals to this enterprise.

Among the results of this research, an important finding is highlighted, uncommon in studies already published, which concerns the feelings experienced by the professionals involved in this process, whether the most experienced or the youngest.

Although the willingness of the group studied to undertake a leadership succession project is clear, it is evident that this is a process that involves an emotional dimension little studied in the literature. This aspect is crucial for the success of such a project, as it depends on the establishment of positive relationships between the various generations of professionals involved, among other aspects.

This gap is possibly due to the fact that, although the knowledge produced about the succession process of Nursing leaderships has progressed over the years, most of the studies focus more specifically on their theoretical and technical aspects. This limitation may also be due to the fact that there are not yet robust studies that show the impact of the implementation of the succession process of Nursing leaderships and their results on institutional indicators.

Most of the studies are still of a theoretical nature, they provide theoretical bases and tools for their implementation. Some studies show good results from leadership development projects, contributing good ideas on how to plan, execute and evaluate such projects.

When considering the Brazilian reality, studies or projects are not known that deal with the development of succession processes of Nursing leaderships in any scenario in the health area. In this sense, this study has an innovative character for bringing the theme to the discussion and to instigate other institutions and / or other researchers to dedicate themselves to the theme. It is believed that this is a starting point for the deepening of the succession of leadership in Nursing in Brazil and sensitization of the relevance of the SP to favor growth and help ensure the future of Nursing.

It is worth highlighting that the data of this research made possible the development of a planning for the succession of leaders, using the appreciative investigation as a method of research and planning, whose results are being prepared for dissemination.
